# A Smartphone App and Cloud-Based Consultation System for Burn Injury Emergency Care

**DOI:** 10.1371/journal.pone.0147253

**Published:** 2016-02-26

**Authors:** Lee A. Wallis, Julian Fleming, Marie Hasselberg, Lucie Laflamme, Johan Lundin

**Affiliations:** 1 Division of Emergency Medicine, Stellenbosch University, Stellenbosch, South Africa; 2 Department of Public Health Sciences, Karolinska Institutet, Stockholm, Sweden; 3 Stellenbosch Institute for Advanced Study (STIAS), Wallenberg Research Centre at Stellenbosch University, Marais Street, Stellenbosch 7600, South Africa; 4 University of South Africa, Preller Street, Pretoria 0002, South Africa; 5 Institute for Molecular Medicine Finland (FIMM), University of Helsinki, Tukholmankatu 8, FI-00290, Helsinki, Finland; University of Florida, UNITED STATES

## Abstract

**Background:**

Each year more than 10 million people worldwide are burned severely enough to require medical attention, with clinical outcomes noticeably worse in resource poor settings. Expert clinical advice on acute injuries can play a determinant role and there is a need for novel approaches that allow for timely access to advice. We developed an interactive mobile phone application that enables transfer of both patient data and pictures of a wound from the point-of-care to a remote burns expert who, in turn, provides advice back.

**Methods and Results:**

The application is an integrated clinical decision support system that includes a mobile phone application and server software running in a cloud environment. The client application is installed on a smartphone and structured patient data and photographs can be captured in a protocol driven manner. The user can indicate the specific injured body surface(s) through a touchscreen interface and an integrated calculator estimates the total body surface area that the burn injury affects. Predefined standardised care advice including total fluid requirement is provided immediately by the software and the case data are relayed to a cloud server. A text message is automatically sent to a burn expert on call who then can access the cloud server with the smartphone app or a web browser, review the case and pictures, and respond with both structured and personalized advice to the health care professional at the point-of-care.

**Conclusions:**

In this article, we present the design of the smartphone and the server application alongside the type of structured patient data collected together with the pictures taken at point-of-care. We report on how the application will be introduced at point-of-care and how its clinical impact will be evaluated prior to roll out. Challenges, strengths and limitations of the system are identified that may help materialising or hinder the expected outcome to provide a solution for remote consultation on burns that can be integrated into routine acute clinical care and thereby promote equity in injury emergency care, a growing public health burden.

## Introduction

Major improvements in burn prevention and care that have been seen over the last decades have mainly benefited those living in high-income countries, and burn mortality rates remain unacceptably high in low- and middle-income countries [1]. Although some of those countries have burn centres, most victims do not get rapidly diagnosed and treated. Novel mobile health (mHealth) based solutions could help remedy this huge public health problem by enabling accurate and timely transmission and interpretation of medical information and rapid interaction between the point-of-care and remote experts.

Studies demonstrate that remote expert diagnosis in clinical areas like radiology, dermatology, neurosurgery, and acute care can be accurate and thereby support clinical decision making at point of care [2–4]. The use of smartphones allows telemedical solutions to be introduced at the point-of-care that include image capture, transmission and receipt, independent of major infrastructure at either end, to any location within range of a mobile phone signal [5, 6]. Burns in particular, because of their relatively superficial and visual nature, are a most suitable target for mHealth applications [7, 8]. Studies have shown that, for example, assessment of the total body surface area (TBSA) [9, 10] and burn depth [10, 11] based on digital images captured of the wound area is feasible. In addition, the recent substantial improvement of mobile phone cameras is likely to facilitate the clinical application of wound photography especially in a telemedical setting [12, 13]. Remote evaluation of burn injuries by experienced physicians has been shown to be more precise and correlate more closely with face to face assessment than estimates by less experienced health care professionals at the point-of-care [6, 13].

Recently, a number of software applications (apps) for smartphones related to burns have been published on Google’s Android and Apple’s iOS store and recently reviewed [14]. Most of the applications are calculation apps for estimating TBSA and total fluid requirement (TFR) [14]. The user interface of a smartphone can, for example, allow the user to mark the injured surfaces on a body diagram. A three dimensional avatar can potentially provide more accurate estimations than conventional visual inspection [15]. For remote consultation to be successful, adequate assessments of the burn location, extent (TBSA), and depth should be enabled since important management decisions are based upon these factors. Decisions include whether transfer to a burn unit is indicated, what fluid resuscitation is required, whether surgery is indicated, and what dressings might be most appropriate [16].

A project has been initiated in the Western Cape of South Africa, where an mHealth application has been developed in order to facilitate rapid diagnosis and adequate care of patients with acute burns. Key components of the solution include a smartphone based application, a server-side system for relay of information between the point-of-care and burns specialists (herein called tele-experts), a web-interface, as well as storage of data and pictures. For the purpose of implementation of the mHealth application a group of dedicated tele-experts have been recruited and are committed to providing advice for each consultation request submitted from the point-of-care. Here we present the technical components of the system, describe the clinical and image data collected by the use of the app, and discuss how we plan to assess its clinical impact (e.g., triage, patient management).

## Methods

### General design of the system

**Fig 1:**
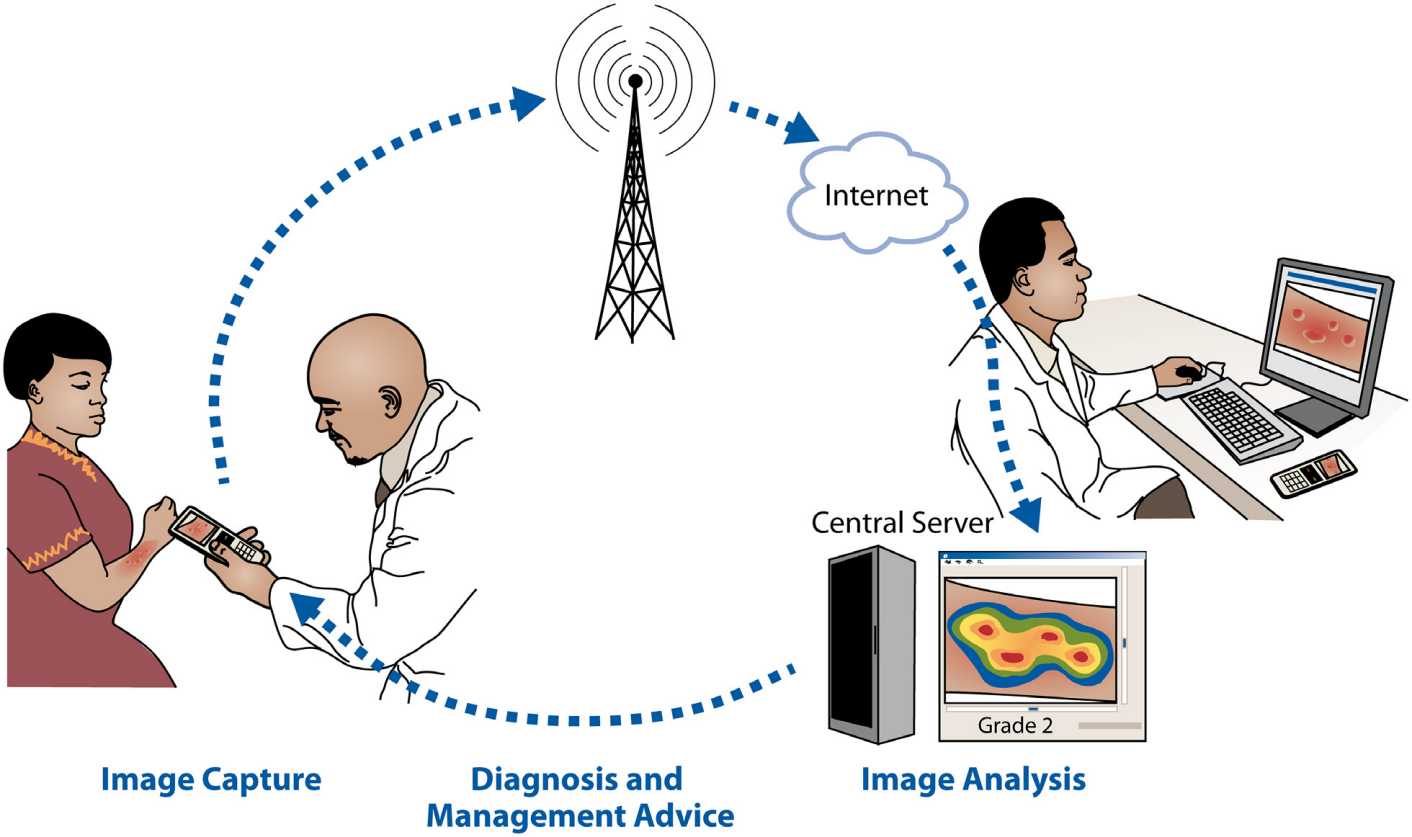
Overview of the burn injury diagnostics and decision support system.

The system is presented in Fig 1. It is designed to store user information and patient data on a network server, to respond to requests on user data from the smartphone client, to receive data (patient data and images) from the mobile phone, respond with defined treatment decision support, relay patient data and images to the burn expert and transfer decision support back to the health care professional in the field. Also, the system is designed to allow for server-side automated image processing (e.g. for image analysis-based assessment of burn severity and depth [17–19]) if robust algorithms for decision-support would become avialable in the future.

### Software application and mobile phone platform

The current version (MoburnZA v. 1.25) of the app is a hybrid between a browser-based (HMTL5 and JavaScript) and a native application for a specific operating system (Android 4.1 or later, Google Inc, Mountain View, CA), developed as a commissioned project by a software company (Pajat Solutions, Espoo, Finland). For the application prototyping and the implementation study a mobile phone (Samsung I8190 Galaxy S III mini, Samsung Electronics Co. Ltd, Suwon, South Korea) equipped with a camera (5 megapixels, 2592 х 1944 pixels, autofocus, aperture f/2.6, focal length 3.54 mm), LED flash and a capacitive touchscreen (4.0 inches, 480 x 800 pixels, Super AMOLED, 16M colours) was used.

### Picture acquisition

For picture acquisition the software application accesses the device camera and allows the user to capture photographs of the burn injury and, to add information on the body part represented in the picture. The default camera settings are activated, but the user is allowed to change the settings and for example, use the flash if needed. A photo viewer is included in the app; a zoomed in view of the captured area is activated when viewing the photos.

### Total body surface area calculator

Calculation of TBSA is done within the application by a dedicated sub-application that allows the user to “paint” areas burned onto an on-screen avatar. Depth of burn is selected by the user (Full Thickness, Partial Thickness, and Indeterminate) which corresponds to different colours on the avatar to allow ease of reference for the user. This is reflected in an on screen “colour key” that is visible whilst using this sub-application. After selecting the depth of burn, the user will select the body part affected by pressing the corresponding area on the avatar. This will in turn zoom into the area to allow for a more accurate “painting” of the area. The user may select anterior or posterior views of the avatar to allocate burn areas to. Multiple burned areas may be selected, as well as multiple depths of burn.

TBSA is then calculated through the allocation of burned area to pixel count painted within the application and is related to the age of the patient provided in the early demographic information provided by the user (this is based on the Lund and Browder Chart, which assigned relative percentages to body areas based on the age of the patient—image attached). The application normalises the painted area such that no areas may be “painted” outside the avatar to limit the overestimation of TBSA through user error. The TBSA is then displayed to the user and utilised to determine resuscitation fluid requirements, utilising the Parkland Formula (Volume of fluid [ml] = 4 (Mass [kg]*Body Area Burned [%])). The user is then instructed to administer 50% of the fluid in the first 8 hours and the remaining 50% in the subsequent 16 hours.

### The cloud-based server application and web-interface

The backend is installed on a server connected to the internet, with 128-bit encryption and using transport layer security (TLS) 1.2. The server software was programmed in an open-source, cross-platform runtime environment (node.js; http://nodejs.org) and installed on a server instance (Ubuntu 12.04.5 LTS, GNU/Linux 3.2.0-40-virtual x86_64), running on a cloud server (Amazon Elastic Compute Cloud, Amazon.com Inc, Seattle, WA) with 1 processing core (Intel(R) Xeon(R) CPU E5-2650 0 @ 2.00GHz), 2 gigabyte random access memory (RAM) and 10 gigabytes of storage.

The web user interface design includes separate views for health care professionals, tele-experts and administrators (described in Results, below).

Data and pictures captured by the mobile application are transferred to the cloud server over a hypertext transfer protocol (HTTP) and stored in a cross-platform document-oriented database (Mongodb, MongoDB Inc, New York, NY) located in a cloud storage environment (Amazon Simple Storage Service, Amazon.com Inc, Seattle, WA). Alerts between the health care provider and the burn expert are sent using a short message service (SMS) gateway (Clickatell, Cape Town, South Africa).

### Data dictionary

In the design of the data input for the application care was taken to search standard terminologies (e.g. ICD, SNOMED, LOINC) for terms to be used. If no standardized coding was found for a data input variable, available terms and classifications were selected from case forms currently used at the study centres or from the literature. The demographic and clinical data currently collected for each patient include: age, weight, pre-existing conditions (e.g. selected chronic diseases, pregnancy), as well as cause and time of the burn. A separate data dictionary with detailed information on the variables and coding has been created and made publicly available (S1 Document).

### Data storage and management

Patient identifiers (name, date of birth, address) are replaced by a reference number when entering data through the mobile phone application to safeguard confidentiality. The reference number is kept in the hospital record and the patient identity is therefore only accessible to the health care provider.

Burn experts and health care professionals (HCPs) at point of care only have access to cases that they have been managing. In the event of a burn expert or HCP having to hand over the patient to another expert or HCP, the system transfers rights to the new HCP or expert, such that security and continuity of care is maintained.

All data are stored on password protected secure databases to ensure participant confidentiality and the current app complies with patient confidentiality rules within South Africa, where it is currently being developed.

### Patient consent and ethical considerations

During the pilot study phase, all patients who will have information transmitted electronically for advice (telemedicine) complete an informed consent document including benefits, risks, a brief description of the services, and signed consent. The planned pilot study has been reviewed and approved by an institutional review board (Health Research Ethics Committee, Stellenbosch University, South Africa, protocol number N13/02/015) and relevant permissions for research at a primary or secondary healthcare facility permission will be obtained from the relevant authorities (Western Cape Department of Health and/or City Health) to conduct the research as stated in the protocol before the pilot study begins.

Separate workflows are designed for a) adults b) children less than 18 years of age but greater than 6 years of age, and c) children aged 6 years and younger (S1 File).

## Results

**Fig 2:**
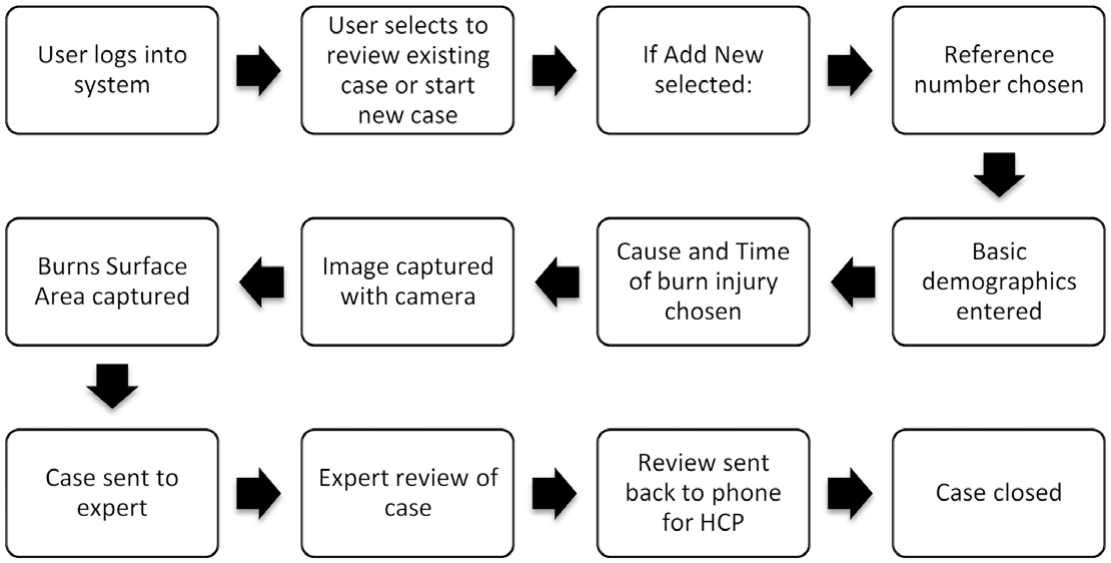
Application workflow for the health care provider.

The mobile application is designed to take the user through a linear workflow in order to collect the required information for transmission to the burns expert (Fig 2). All communication takes place through the application—i.e. comments and advice are received and viewed through the application, such that no other software is required to manage the patient.

### The mobile application user interface

The current version of the application is designed for one of the major mobile phone platforms (Android 4.1 and later, Google Inc, Mountain View, CA) and run by clicking the application icon.

#### User Registration

**Fig 3:**
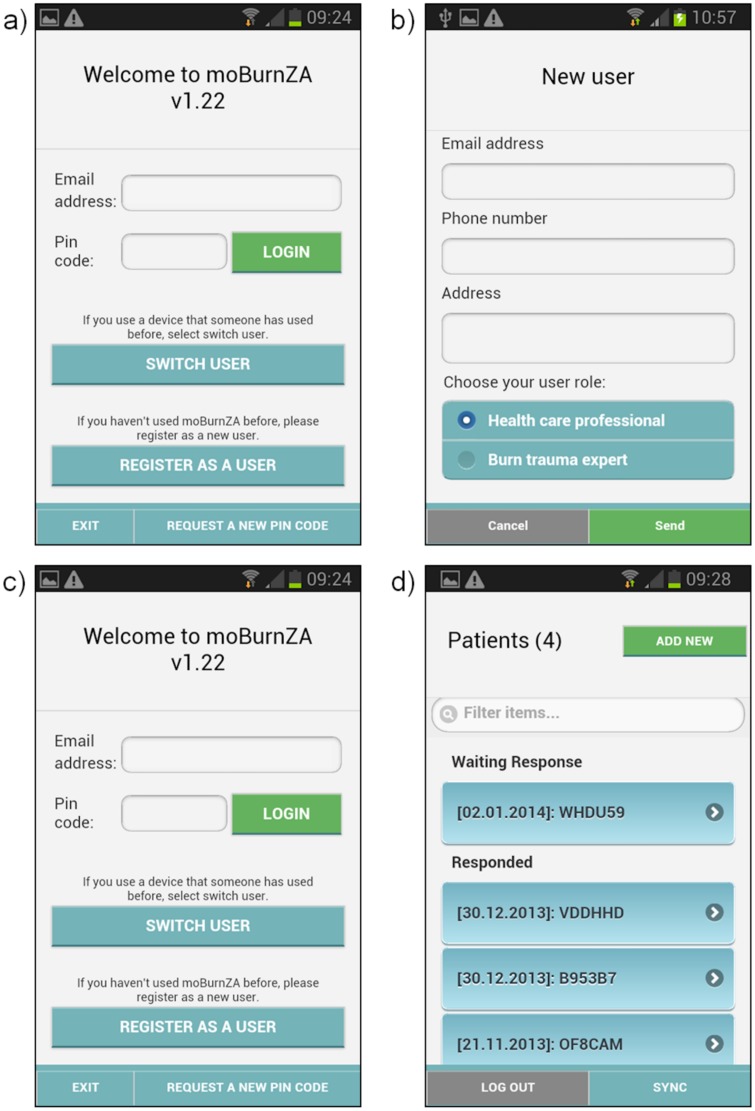
User Registration (a-b), selection of role (b), login (c) and list of cases according to status (d).

Prior to using the application for the first time, a user must register on the system. This is to protect confidential information and prevent unauthorized use of the application. Registration should occur at the training session, but can be done through the application as well (Fig 3a and 3b). Only recognized and authorized users are allowed to register on the application and approval for the registration is done by the system administrator. Registration may also be done remotely by an administrator (this is the process that is followed during training).

The application is designed to lead users through data entry as simply as possible, gathering all the required information as quickly as possible so as to minimise patient care flow. Many of the buttons can be seen but not pressed until a previous entry is made to prevent any critical information being missed.

#### User login

Using the details provided through the registration process, the user enters login details to gain access to the application (Fig 3c).

#### Enter a case

The user then has the option to review existing cases (either those that have received Expert Feedback or those still awaiting feedback) or add a new case (Fig 3d).

#### Enter reference number

**Fig 4:**
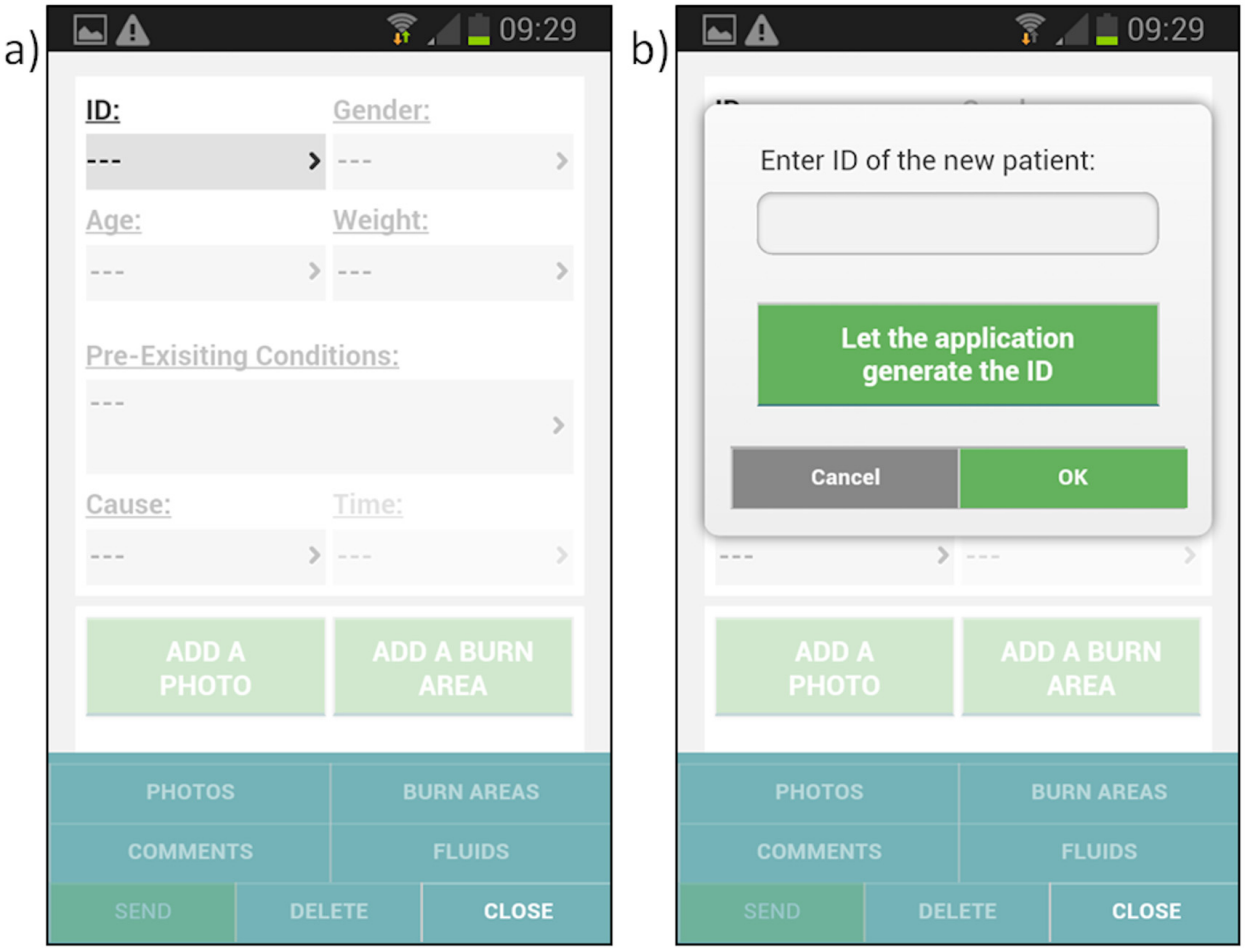
Entering a reference number to identify the injured person.

The user must then choose a unique reference number for the patient. For the purposes of the pilot study and to ensure patient confidentiality, a random identifier can be generated by the application (Fig 4).

#### Demographic information

**Fig 5:**
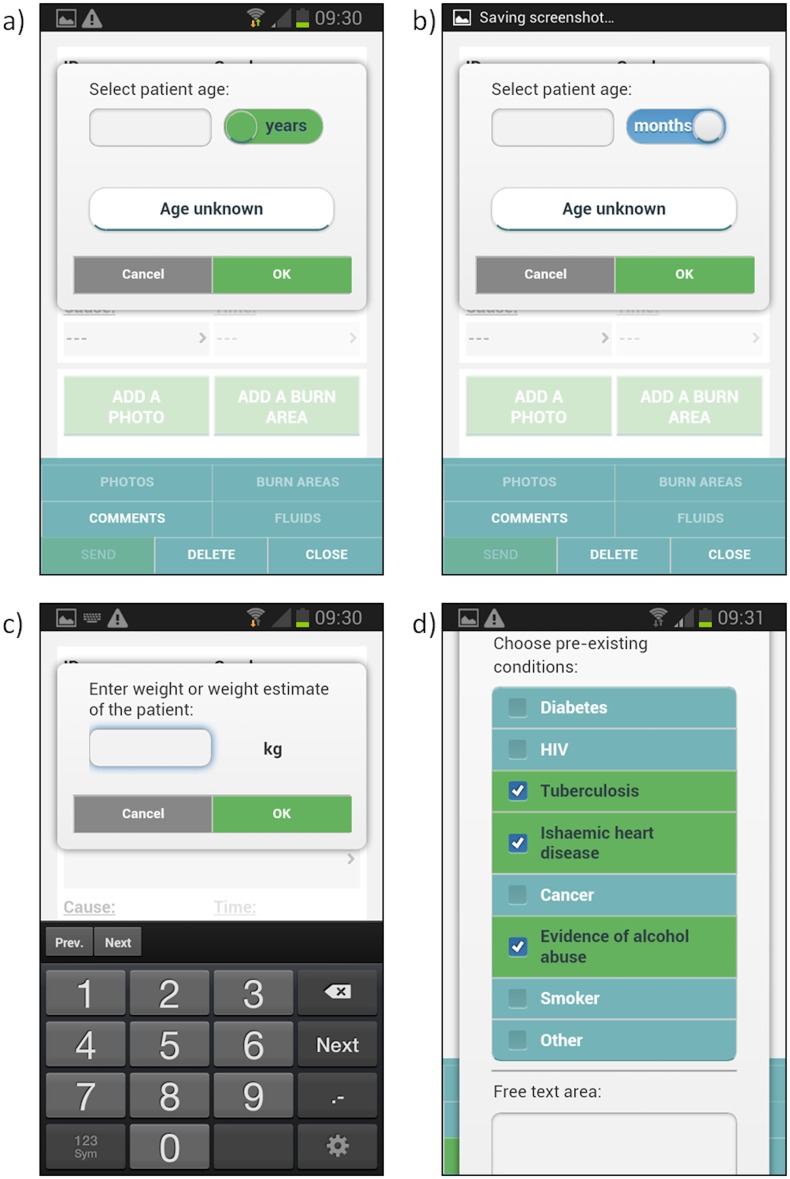
Demographic information a) patient age in years, b) age in months in case of less than one year old patient c) patient weight, d) pre-existing conditions.

**Fig 6:**
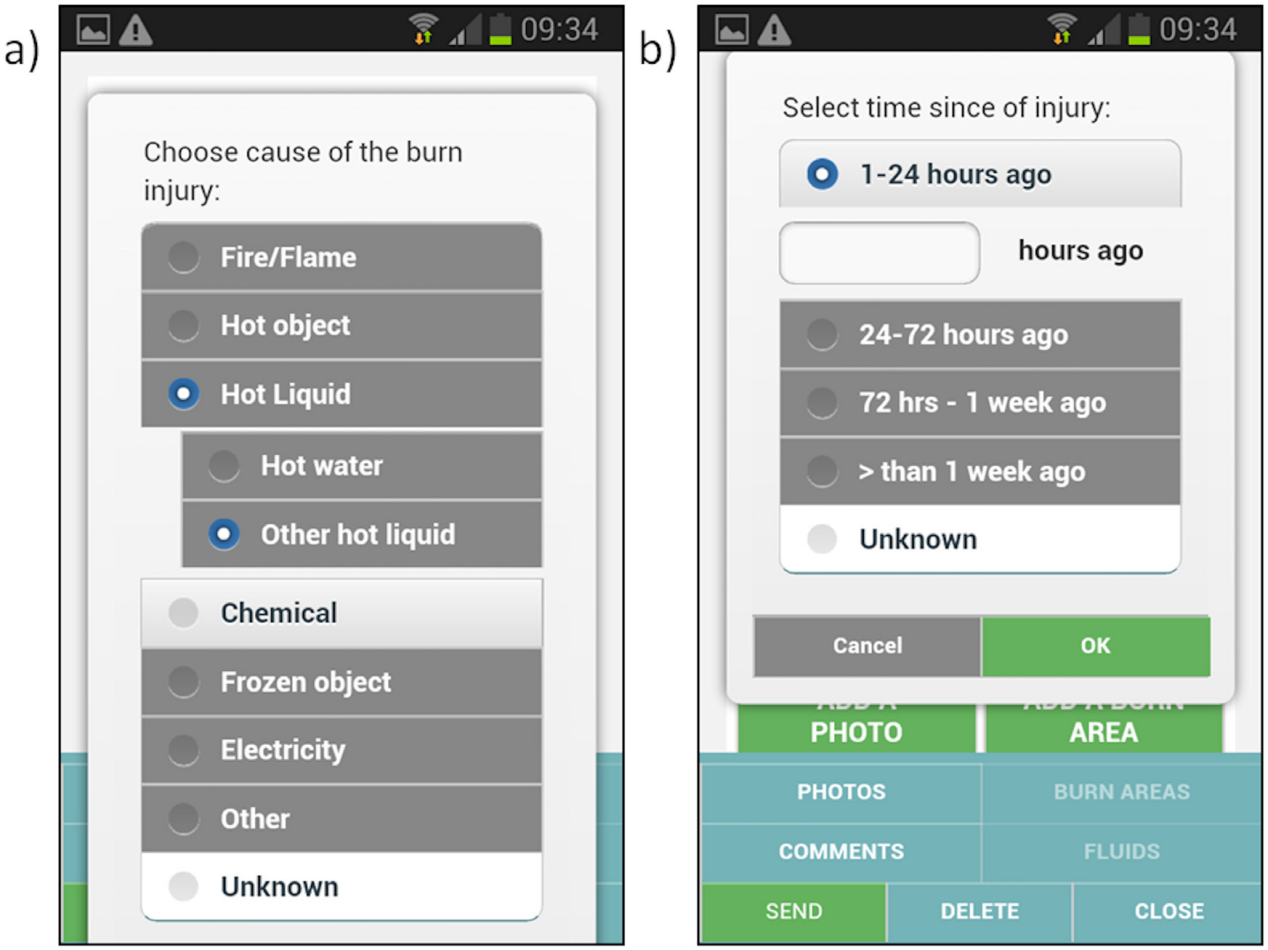
a) Cause and b) time of the burn injury.

The user then enters basic demographic information, such as gender, age, weight and pre-existing medical conditions (Fig 5a–5d), as well as injury information such as cause and time of injury (Fig 6). This information will assist the burn expert in providing advice to the user as well as ensure a comprehensive clinical record of the injury.

#### Capture photos

**Fig 7:**
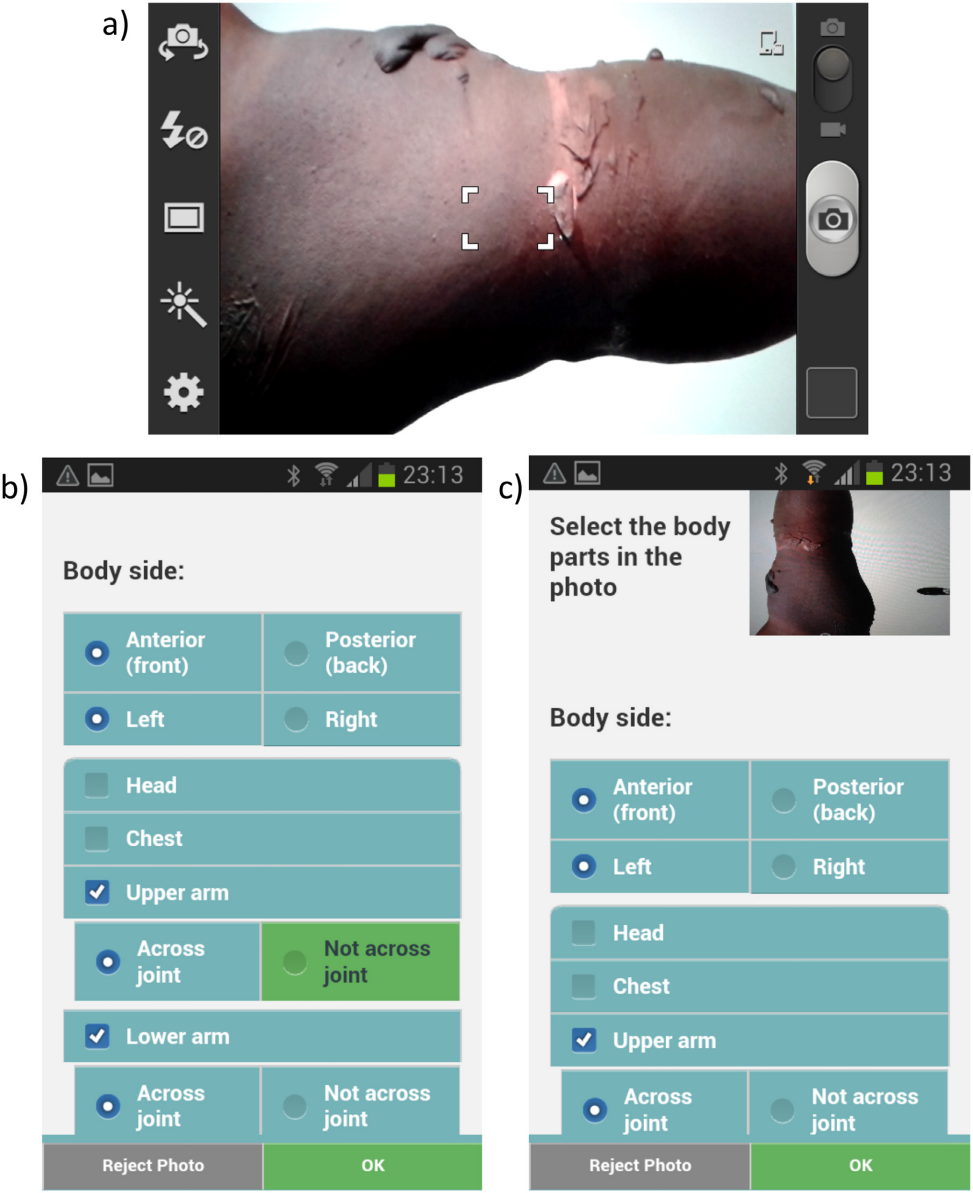
a) Taking a photo of the burn injury and b) input of burn location on the body.

The user can then take photographs of the injury by using the built in camera and select location of the wound from a body part category index (Fig 7). Photographs assist the burns experts significantly in making a diagnosis and providing advice [10]. As part of the training process the user receives guidance on how to capture pictures of the burn wounds (Table 1).

**Table 1:** When taking a photograph of the burn injury, the following tips are given to the user as part of the training process.

**1**.	Remind the patient why you are taking the photograph and that the image will be sent remotely to a burn expert. The patient can still withdraw from the study at this point if they wish.
**2**.	Use the camera flash if needed. This will ensure that the injury is well lit regardless of the lighting in the room.
**3**.	Try and take the photograph perpendicular to the burn injury—this will help ensure that the flash lights up the injury evenly.
**4**.	Take as many images as you need to represent the burn injury. You may wish to take images from different heights or angles to capture all the information you need.

#### Painting burn area

**Fig 8:**
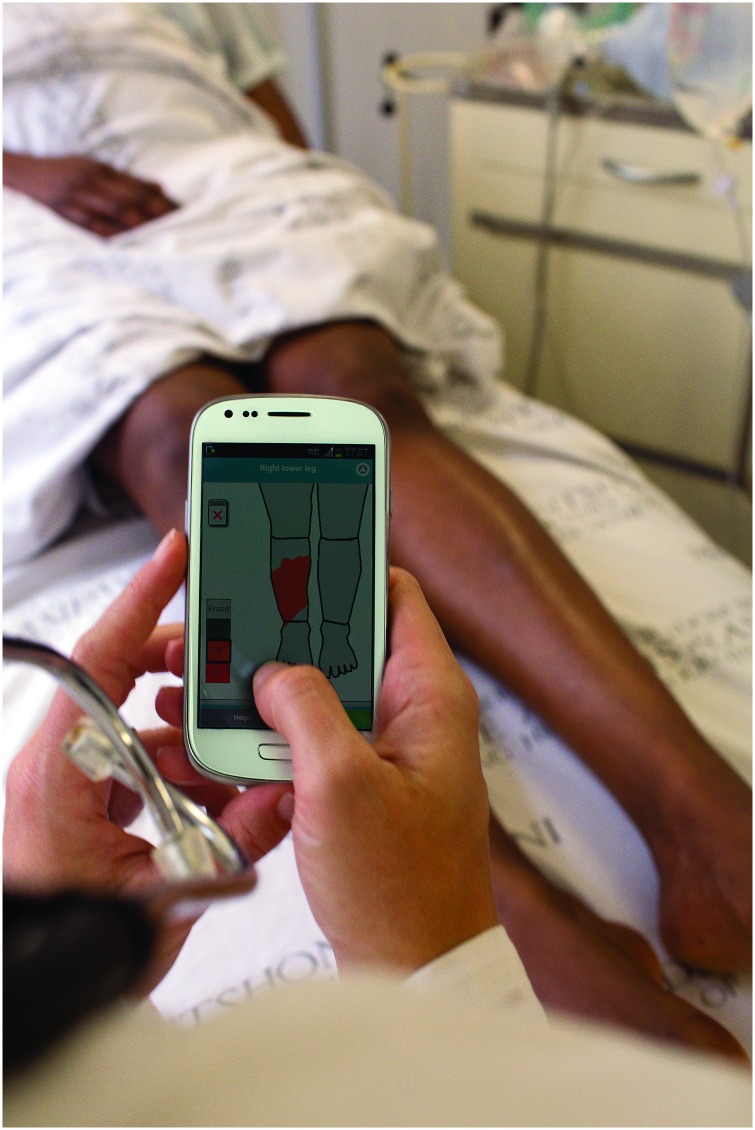
The app in use.

**Fig 9:**
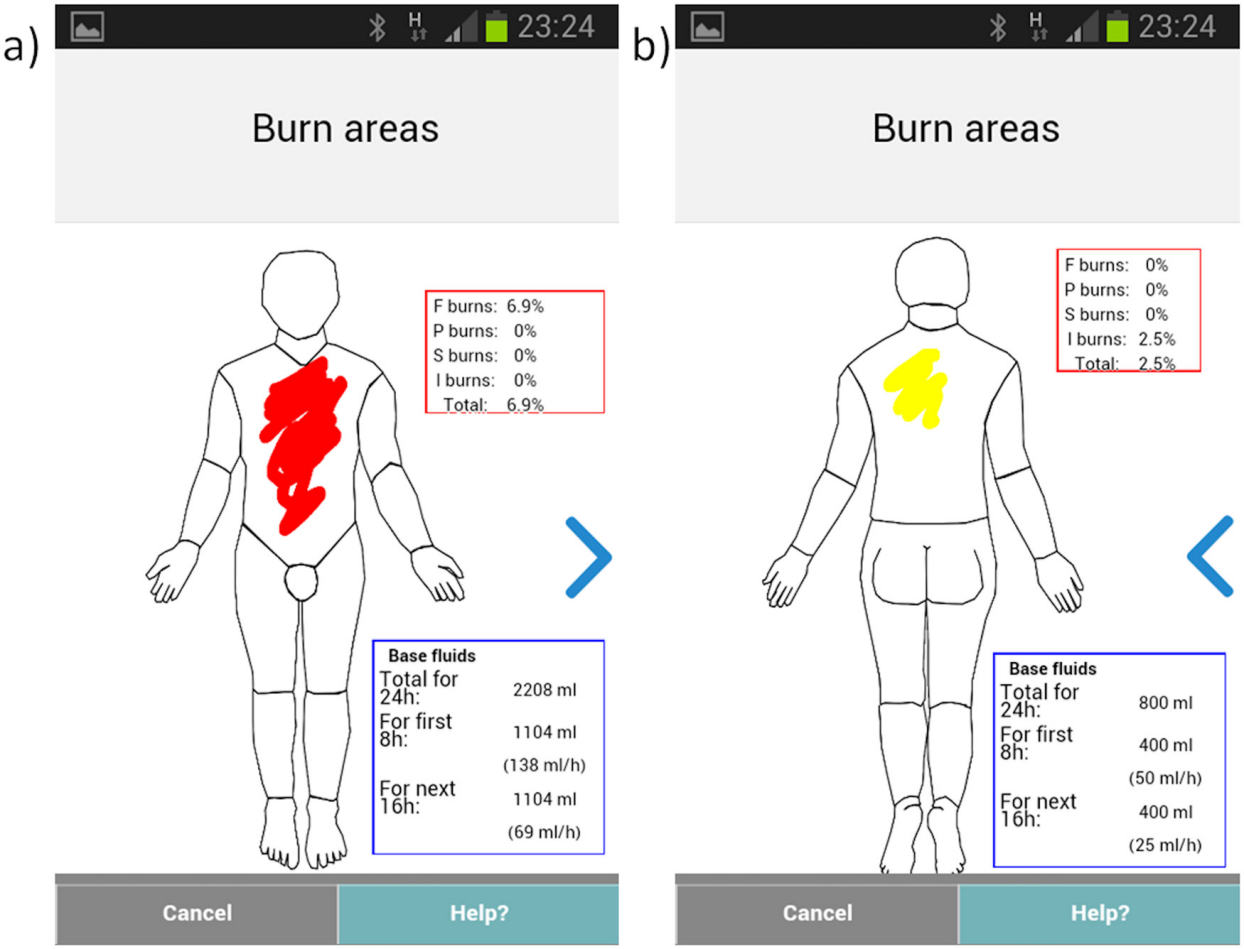
The Total Body Surface Area (TBSA) calculator that includes a a) front and b) back body diagram and a touch interface for indicating the location, depth and extent of the burn injury.

The application then directs the user to select the depth of burn and to paint the burn injury area using a finger on a body map schematic (Figs 8 and 9); this information is linked to the corresponding body part categories and body side.

#### TBSA Calculator

**Fig 10:**
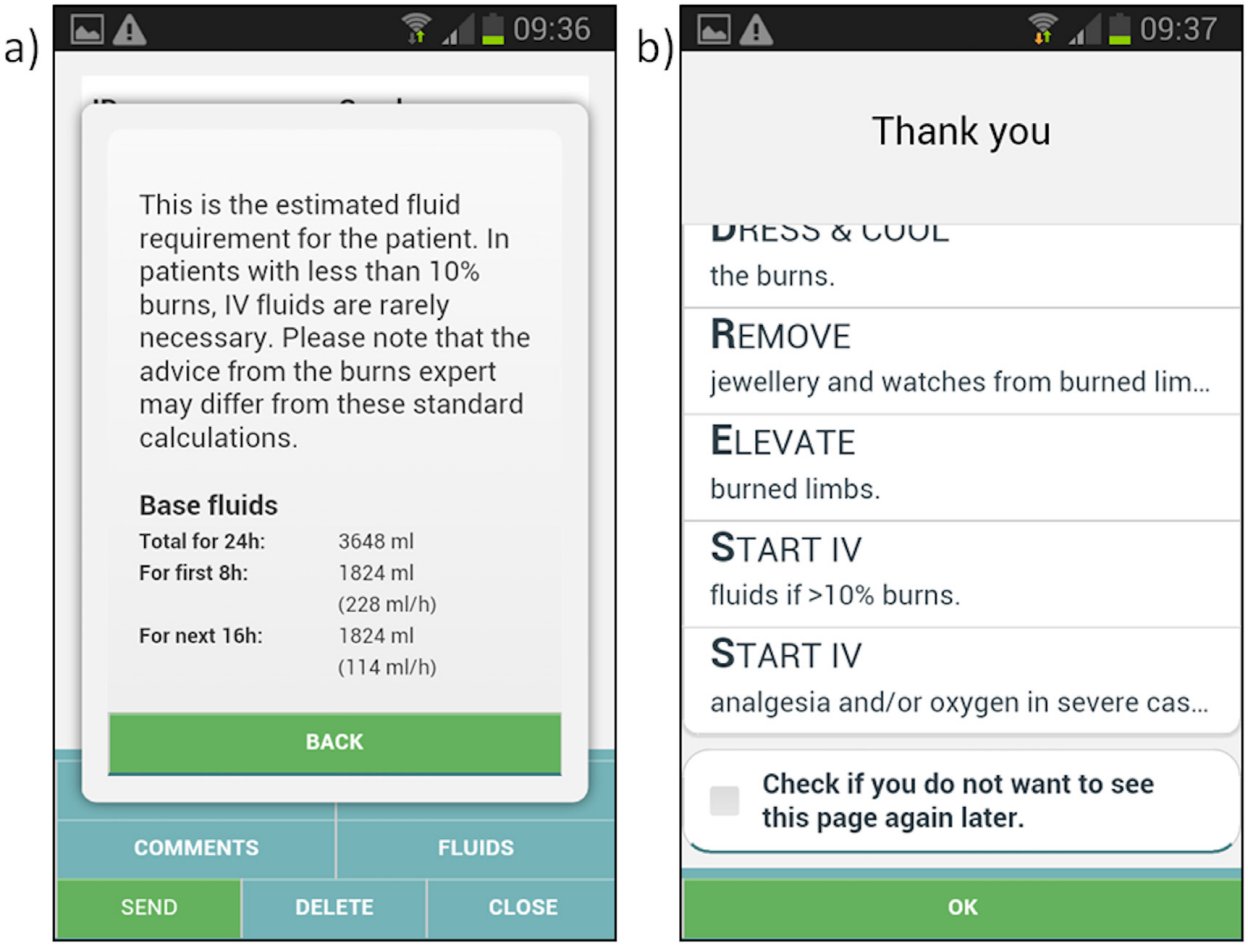
Treatment advice based on data input. a) Estimated fluid requirements calculated according to the Parkland formula based on the total body surface area b) general advice for burn treatment.

Based on the painted area of the burn the calculator will automatically calculate the TBSA and hence suggested intravenous fluid volume (using the modified Parkland formula [20]). The application suggests three depth categories for burn injuries with corresponding advice for coding presented in the user manual (Table 2). Also, the app provides immediate basic treatment advice based on the entered information and calculated TBSA (Fig 10).

**Table 2:** Depth categories for burn injuries and guidelines for the categories as presented in the application manual.

1.	**Superficial Thickness Burns**
	This is similar to sunburn and is the mildest form of burn. There may be blistering. These burn areas are not included in fluid calculations, but should be recorded for the clinical notes.
2.	**Partial Thickness Burns**
	These burns affect the epidermal layer and usually present as painful, reddened and sometimes blistered areas on the body.
3.	**Full Thickness Burns**
	These burns have burned beyond the dermis and are unable to heal themselves. They are usually white/grey and feel leathery to the touch. These burns require a skin graft.
4.	**Indeterminate Depth Burns**
	These burns are difficult to assess at the time of injury and lie between superficial and full thickness burns. Time is needed to see if these burns progress to full thickness or remain superficial and heal spontaneously.

#### Interaction with burn expert

The application allows the health care provider to interact with tele-experts in real time to get advice and facilitate referral to the correct facility. The application has an integrated communication system built in that seamlessly transmits the case to the burn expert and also allows for advice to be sent back to the phone. There is also a short message service system built in to allow for two-way communication.

#### The tele-expert interface

**Fig 11:**
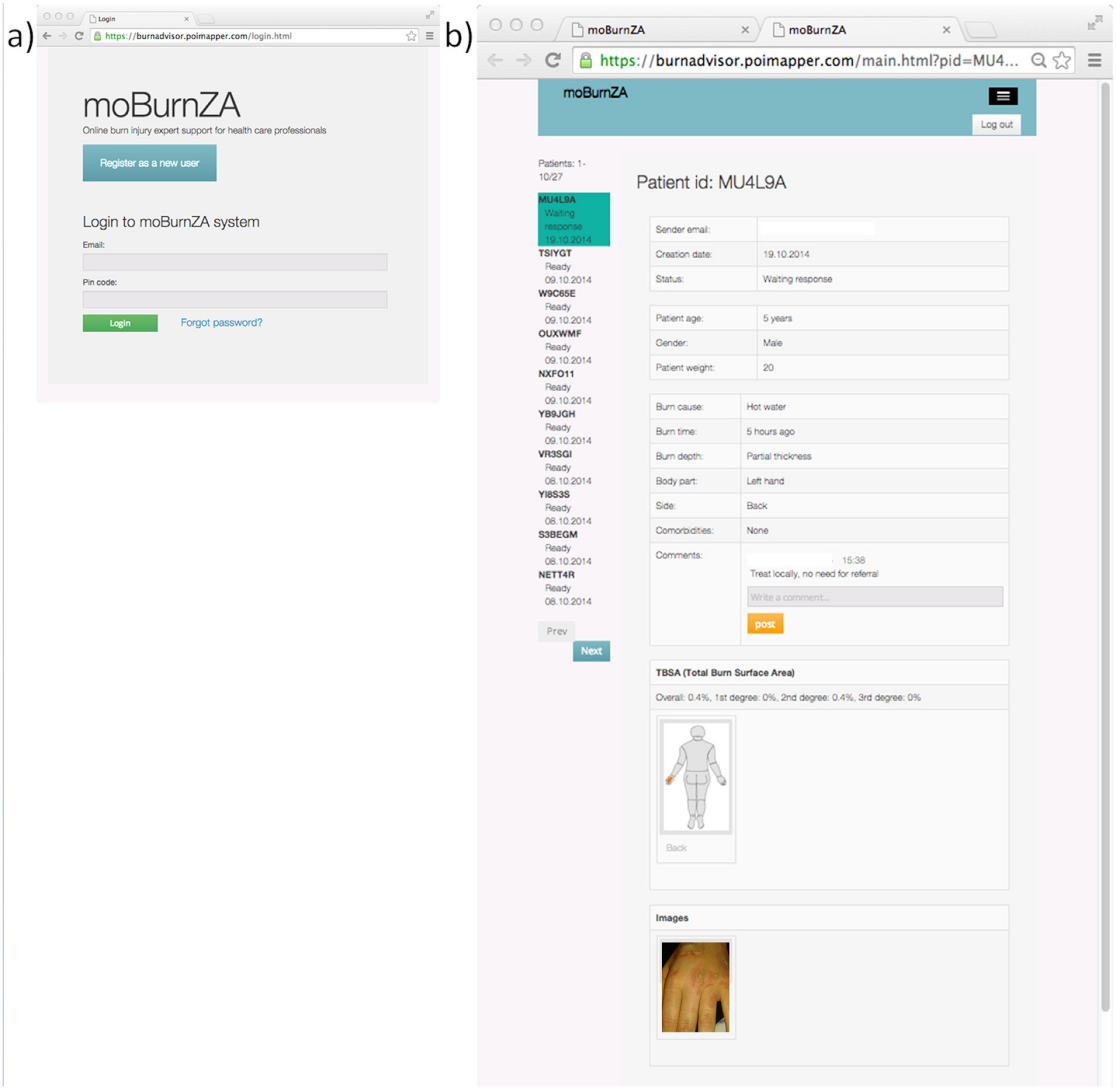
Web login and user interface for the burn expert.

The burn expert can access the system either through the mobile application or a web interface (Fig 11). When logged in, the burn expert is presented with a list of pending cases to select from: all data collected with the app, the painted schematic body figure, and the photos are presented to the tele-expert (Fig 11). By clicking on any of the pictures, an enlarged (1:1) version is displayed. The interface includes pre-defined options for advice, as well as freetext box where the burn experts enters specific advice and comments.

#### Treatment advice

When the case has been reviewed and commented on by the Burn Expert, the user will receive an SMS and email from the system informing the user that the case has new advice.

**Fig 12:**
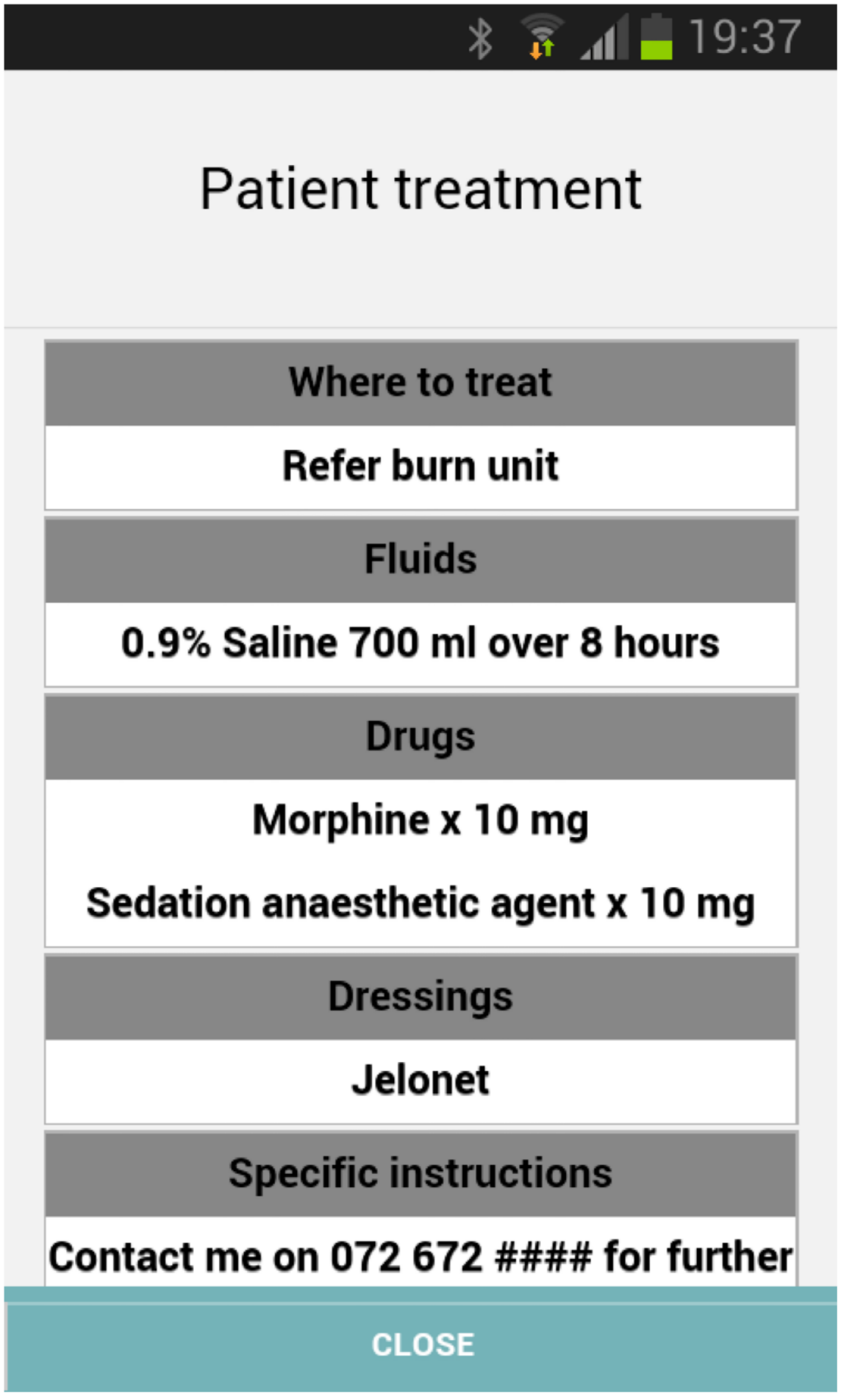
Treatment advice provided by the burn expert.

Patient related advice includes: a) Where to treat (and whether to transport) b) What fluids to administer c) What medication to administer d) What dressings to apply e) Other specific instructions (Fig 12)

The user can also send additional comments to the Burn Expert by entering this information in a field.

Logging out of the system ensures that no unauthorized person can access the records on the smartphone. The system performs an auto logout after 5 minutes, but users are also advised to logout of the system whenever they no longer need to use it.

## Discussion

Timely clinical assistance to injury diagnostics and patient management is a challenge in resource poor settings. We expect that the system will provide a solution for remote consultation in acute burns that can be integrated into routine clinical care in the emergency setting and discuss how the method will be implemented.

There are a number of software applications for mobile phones i.e. apps intended to support management of burn injury patients, such as the BurnCare app by Pierre-Antoine Meley (for Android operating system), BurnMed Pro by Johns Hopkins Mobile medicine (for iOS), Medrills:Burns by ArchieMD Inc (iOS), LiAo Burns by Omesoft (for iOS), uBurn by JAMB Innovations, LLC (iOS), MerseyBurns by St Helens and Knowsley Teaching Hospitals NHS Trust (Android) and Rapid Burn Assessor and BurnCase 3D by RISC Software GmbH (iOS) [14, 15]. As far as we can determine, very few of these have been validated or described in the scientific literature, with the exception of the uBurn, MerseyBurns [21, 22], Rapid Burn Assessor and BurnCase 3D apps [15, 23]. Typical functionalities of the available apps for burn injury care include calculation of TBSA and estimation of TFR. Our approach differs from the available ones in that it provides access to actual expert consultation in addition to the TBSA and TFR. The solution that we describe, to be implemented at first in the determined geographic area, includes the whole consultation process, with registered tele-experts that are committed to respond to consultation requests and a central server that handles and store the pictures and data.

In planning the current solution together with the software development company, we had to make a number of decisions regarding the general design. Those include selection of operating system, native versus browser-based application, database, cloud platform, sms gateway and trial mobile phone device. The overall aim was to create a solution that can be implemented in the determined geographic area, but also allows transfer of the method to various settings and provides interoperablity through the use of controlled vocabularies for data coding. An effort was made to minimize the number of input variables to make data capture as brief and efficient as possible and to utilize the touch interface for entry of wound location through a painting tool. The information flow is designed to be in accordance with general burn case management at the point-of-care. The app allows the user to directly connect a picture of the burn wound with a corresponding list of body parts, to ensure that the burn expert can verify the wound locations in the photographs. The user can also change some of the camera settings, such as flash and pixel dimensions. However, according to feed-back from a panel formed of the experts enrolled in the project, it might be needed to control these parameters more strictly in future versions to minimize variability of the image quality. On the other hand, most of recent smartphone cameras capture photos of good quality [13] and have been reported to provide acceptable quality for burn wound assessment [12, 13].

In order for staff to feel at ease with the system, they will receive in-depth prior training; in addition, they will be provided with on-going support—the system uptake will be closely followed up to make sure that any problems are dealt with promptly. Staff training for using the system will be designed taking into consideration the different level of experience with smart phones. Anxiety of using mobile applications is an issue that must be considered and the training should be designed to empower the staff and not intimidate.

This system represents an addition to normal care; the clinical advice delivered through the system and app is intended for burn care advice and can be ignored by the treating clinician at their discretion. In line with all other clinical expert advice, the front line treating clinician retains responsibility for the actualclinical care delivery.

Anonymity of the images will be strived for but can be hard to achieve for patients with burns on the face or head. The images and clinical information will be treated as confidential, be assigned a unique identifier unrelated to any other patient identifiers, and will be stored on a secure cloud based server which is accessed through a password protected personal account for on-call tele-experts and for study personnel. No data will be held on the phones—all data will be deleted automatically once the server has received it as an upload. Any data which is not successfully uploaded will be remotely wiped after 24 hours. Tele-experts and treating clinicians will only have access to the cases which they are directly responsible for.

Technical support for the server will be provided by the development company. Only the involved health care personnel, the experts and the staff employed in the project will have access to the data and images.

The use of the consultation system will potentially add up to 2–3 minutes to the clinical consultation (the exact time will only become clear once the application is fully developed and tested). However, the application is expected to improve care delivery and facilitate local management whenever possible, which reduces stress of transport and long distances to travel for patient and caregivers. Additionally, care is likely to be enhanced through the timely addition of specialist advice and knowledge through the telemedicine platform.

Burn specialist care through telemedicine has the potential to reach those living in underserved areas—frequently out of reach of specialist care—and therefore contributing to a more just/equitable distribution of health care. The app is intended to be free to use in low income settings, whereas in higher income settings users will pay a charge for the app. The mHealth application will follow the regulations and guidelines for development and application of health apps [24] and respect of patient security and autonomy will be emphasized. If any other disease or disorder would be suspected, referral to appropriate department will be made.

The technical performance of the system during implementation will be measured using a standard set of data either gathered during the teleconsultation or generated by the application every time there is a teleconsultation: a) measures relative to the use of the application at the point-of-care (time of composition, submission, number of pictures) b) measures of tele-expert (response time, eventual request of complementary information, place of response) c)measures of the patient/injury (age, burn site, burn depth and TBSA) d) measures of eventual system failure.

We also plan to have regular contacts with the clinicians at point of care (on site or by telephone) as well as with the tele-experts to capture any additional (positive or negative) aspects relative to the system that they may wish to comment on and that can help improve the system performance.

Throughout the project, the experiences acquired in the participating health care facilities and by the tele-experts will be synthesized and discussed in a series of panel of experts meetings (POEMs).

There are a number of potential limitations of the proposed system. A first one relates to the implementation itself, success of which will be conditional on healthcare personnel buy-in. During the planned implementation, we will also closely monitor the acceptance among the staff and engage in actively marketing and promoting the system, and addressing any concerns which have arisen.

There might also be limitations related to the mobile networks coverage in the region. To address this, the system is designed to function regardless of a short network interruption (e.g. preferably using text messages as they do not need continuous connection). In addition, while waiting for the reply from the expert, the healthcare worker will be provided with some basic general advice (e.g. manage the cervical spine, give oxygen, elevate limbs, etc.) that do not depend on the connection.

There is also a risk for clinical shortcomings. There are good reasons to believe that the clinical accuracy of the system will be good [6, 9, 11–13] and we plan for specific evaluations—and adjustments—of that prior to staff training. For patient management at point of care, staff training and needs inventory will be made in every facility to safeguard that all patients can receive care locally if and when needed.

## Conclusion

Visual inspection of a burn injury provides important information with regard to clinical decision making on treatment and patient referral to specialist burn care. The rapid development and expansion of mobile networks and the global adoption of mobile phones provide effective means for voice, text and image-based consultation. The development of high-speed data networks can especially facilitate transfer of images and therefore applications that utilize these opportunities are needed. The smartphone-based consultation system developed and described in the current study presents a platform that connects the healthcare providers at the point-of-care with the burn experts and has the potential to improve access to burn diagnostics and expert advice.

## Supporting Information

S1 Document Patient Consent. (PDF)

S1 File Data Dictionary. (PDF)

## Abbreviations

User: health care professional, user of the proposed platform and software application
Case: patient, who has suffered a burn injury
App: software application installed and running on a mobile phone

## References

[1] WHO. A WHO plan for burn prevention and care2008 14.1.2015 [cited 2015 14.1.2015]. Available from: http://whqlibdoc.who.int/publications/2008/9789241596299_eng.pdf.

[2] Boulanger B, Kearney P, Ochoa J, Tsuei B, Sands F. Telemedicine: a solution to the followup of rural trauma patients? Journal of the American College of Surgeons. 2001;192(4):447–52. Epub 2001/04/11. . 11294400 10.1016/s1072-7515(01)00796-7

[3] Hasselberg M, Beer N, Blom L, Wallis LA, Laflamme L. Image-based medical expert teleconsultation in acute care of injuries. A systematic review of effects on information accuracy, diagnostic validity, clinical outcome, and user satisfaction. PLoS One. 2014;9(6):e98539 Epub 2014/06/03. 10.1371/journal.pone.0098539 PONE-D-13-46976 [pii] 24887257 PMC4041890

[4] Saffle JR, Edelman L, Theurer L, Morris SE, Cochran A. Telemedicine evaluation of acute burns is accurate and cost-effective. The Journal of trauma. 2009;67(2):358–65. Epub 2009/08/12. . 19667890 10.1097/TA.0b013e3181ae9b02

[5] Keane MG. A review of the role of telemedicine in the accident and emergency department. Journal of telemedicine and telecare. 2009;15(3):132–4. Epub 2009/04/15. 10.1258/jtt.2009.003008 . 19364895

[6] Shokrollahi K, Sayed M, Dickson W, Potokar T. Mobile phones for the assessment of burns: we have the technology. Emergency medicine journal: EMJ. 2007;24(11):753–5. Epub 2007/10/24. 10.1136/emj.2007.046730 17954825 PMC2658315

[7] Rao B, Lombardi A 2nd. Telemedicine: current status in developed and developing countries. J Drugs Dermatol. 2009;8(4):371–5. Epub 2009/04/15. . 19363855

[8] Wallace DL, Hussain A, Khan N, Wilson YT. A systematic review of the evidence for telemedicine in burn care: with a UK perspective. Burns. 2012;38(4):465–80. Epub 2011/11/15. S0305-4179(11)00310-X [pii] 10.1016/j.burns.2011.09.024 . 22078804

[9] Kiser M, Beijer G, Mjuweni S, Muyco A, Cairns B, Charles A. Photographic assessment of burn wounds: a simple strategy in a resource-poor setting. Burns. 2013;39(1):155–61. Epub 2012/06/01. S0305-4179(12)00126-X [pii] 10.1016/j.burns.2012.04.003 . 22647494

[10] Boissin C, Laflamme L, Wallis L, Fleming J, Hasselberg M. Photograph-based diagnosis of burns in patients with dark-skin types: The importance of case and assessor characteristics. Burns. 2015 Epub 2015/02/27. S0305-4179(14)00448-3 [pii] 10.1016/j.burns.2014.12.014 . 25716764

[11] Boccara D, Chaouat M, Uzan C, Lachere A, Mimoun M. Retrospective analysis of photographic evaluation of burn depth. Burns. 2011;37(1):69–73. Epub 2010/08/07. S0305-4179(10)00147-6 [pii] 10.1016/j.burns.2010.05.017 . 20688436

[12] Godwin ZR, Bockhold JC, Webster L, Falwell S, Bomze L, Tran NK. Development of novel smart device based application for serial wound imaging and management. Burns. 2013;39(7):1395–402. Epub 2013/06/19. S0305-4179(13)00098-3 [pii] 10.1016/j.burns.2013.03.021 . 23768708

[13] Boissin C, Fleming J, Wallis L, Hasselberg M, Laflamme L. Can We Trust the Use of Smartphone Cameras in Clinical Practice? Laypeople Assessment of Their Image Quality. Telemed J E Health. 2015 Epub 2015/06/16. 10.1089/tmj.2014.0221 . 26076033 PMC4649724

[14] Wurzer P, Parvizi D, Lumenta DB, Giretzlehner M, Branski LK, Finnerty CC, et al Smartphone applications in burns. Burns. 2015;41(5):977–89. Epub 2015/02/07. S0305-4179(14)00408-2 [pii] 10.1016/j.burns.2014.11.010 . 25655039

[15] Parvizi D, Giretzlehner M, Dirnberger J, Owen R, Haller HL, Schintler MV, et al The use of telemedicine in burn care: development of a mobile system for TBSA documentation and remote assessment. Annals of Burns and Fire Disasters. 2014;27(2):94–100. PMC4396802. 26170783 PMC4396802

[16] Karpelowsky JS, Wallis L, Madaree A, Rode H. South African Burn Society burn stabilisation protocol. S Afr Med J. 2007;97(8):574–7. Epub 2007/10/31. . 17966146

[17] Ba Acha, Serrano C, Acha JI, Roa LM. Segmentation and classification of burn images by color and texture information. BIOMEDO. 2005;10(3):034014–03401411. 10.1117/1.1921227 16229658

[18] Serrano C, Acha B, Gomez-Cia T, Acha JI, Roa LM. A computer assisted diagnosis tool for the classification of burns by depth of injury. Burns. 2005;31(3):275–81. Epub 2005/03/19. S0305-4179(04)00345-6 [pii] 10.1016/j.burns.2004.11.019 . 15774281

[19] Wantanajittikul K, Theera-Umpon N, Auephanwiriyakul S, Koanantakool T, editors. Automatic segmentation and degree identification in burn color images. Biomedical Engineering International Conference (BMEiCON), 2011; 2011 29–31 Jan. 2012.

[20] Baxter CR, Shires T. Physiological response to crystalloid resuscitation of severe burns. Ann N Y Acad Sci. 1968;150(3):874–94. Epub 1968/08/14. . 4973463 10.1111/j.1749-6632.1968.tb14738.x

[21] Morris R, Javed M, Bodger O, Hemington Gorse S, Williams D. A comparison of two smartphone applications and the validation of smartphone applications as tools for fluid calculation for burns resuscitation. Burns. 2014;40(5):826–34. Epub 2013/11/20. S0305-4179(13)00345-8 [pii] 10.1016/j.burns.2013.10.015 . 24246618

[22] Barnes J, Duffy A, Hamnett N, McPhail J, Seaton C, Shokrollahi K, et al The Mersey Burns App: evolving a model of validation. Emerg Med J. 2015;32(8):637–41. Epub 2014/11/06. emermed-2013-203416 [pii] 10.1136/emermed-2013-203416 . 25371408

[23] Giretzlehner M, Owen R, Dirnberger J, Haller H, Kamolz L, editors. Rapid Burn Assessor 15th EBA Congress; 2013; Vienna, Austria.

[24] D4. Regulation of health apps: a practical guide2012 [cited 2015 14.1.2015]. Available from: http://www.d4.org.uk/research/regulation-of-health-apps-a-practical-guide-January-2012.pdf.

