# Supplementary material for: A Smartphone App and Cloud-Based Consultation System for Burn Injury Emergency Care
Source: PLoS One. 2016 Feb 26;11(2):e0147253. doi: 10.1371/journal.pone.0147253 (PMC4769217; doi:10.1371/journal.pone.0147253)
Supplement: S1 Document — (PDF) [file pone.0147253.s001.pdf]

S1 Document:

Patient Consent

Adults are required to complete an adult consent form prior to participation in the research or having their information transmitted electronically using the application. The patient will have the consent form read to him/her and have the contents explained in their own language so that they understand the research completely. The application reference number is recorded on the consent form. The patient will receive the information attached to the consent so that they can read this again in their own time and contact the research team if they have any questions.

For children < 18 years of age and > 6 years of age, the participants are required to complete two forms for consent: 1) a parent/guardian consent form; and 2) a child assent form prior to participation in the research or having their information transmitted electronically using the application. It is important to note that even if the parent/guardian consents to the research taking place but the child does not provide assent, then the research should not take place. It is the child's right to refuse participation at all times. Otherwise, the workflow for this age group is similar to the workflow for an adult.

For children aged 6 years or less the workflow is the same as for the older children, but it is important to note that even if the parent/guardian consents to the research taking place but the child does not provide assent and the child is deemed to be old enough to make that decision, then the patient will not be included in the study.
