# Supplementary material for: A Smartphone App and Cloud-Based Consultation System for Burn Injury Emergency Care
Source: PLoS One. 2016 Feb 26;11(2):e0147253. doi: 10.1371/journal.pone.0147253 (PMC4769217; doi:10.1371/journal.pone.0147253)
Supplement: S1 File — (PDF) [file pone.0147253.s002.pdf]

Variable 1	**User ID**
Name (GUI ENG)	User ID
Name (database)	UserID
UMLS ID	C1708731
Type	String
Length max char	25
Unit	
Group	User_db
Data entry	Autogenerate
Comments	
Terminology	
Index	
Codes:	
Category1	
Category2	
Category3	
Category4	
Category5	
Category6	
Category7	
Category8	
Category9	
Category10	

Variable 2	**User Role**
Name (GUI ENG)	User Role
Name (database)	UserRole
UMLS ID	C1883484
Type	String
Length max char	50
Unit	
Group	User_db
Data entry	Dropdown
Comments	
Terminology	
Index	User_categories
Codes:	
Category1	Health care professional
Category2	Burn injury expert
Category3	Administrator
Category4	
Category5	
Category6	
Category7	
Category8	
Category9	
Category10	

Variable 3	**First Name**
Name (GUI ENG)	First Name
Name (database)	FirstName
UMLS ID	C1443235
Type	String
Length max char	25
Unit	
Group	User_db
Data entry	Textbox
Comments	
Terminology	
Index	NA
Codes:	
Category1	
Category2	
Category3	
Category4	
Category5	
Category6	
Category7	
Category8	
Category9	
Category10	

Variable 4	**Last Name**
Name (GUI ENG)	Last Name
Name (database)	LastName
UMLS ID	C1301584
Type	String
Length max char	50
Unit	
Group	User_db
Data entry	Textbox
Comments	
Terminology	
Index	NA
Codes:	
Category1	
Category2	
Category3	
Category4	
Category5	
Category6	
Category7	
Category8	
Category9	
Category10	

Variable 5	**Email address**
Name (GUI ENG)	**Email address**
Name (database)	**EmailAddress**
UMLS ID	**C1705961**
Type	**String**
Length max char	
Unit	
Group	**User_db**
Data entry	**Textbox**
Comments	
Terminology	
Index	**NA**
Codes:	
Category1	
Category2	
Category3	
Category4	
Category5	
Category6	
Category7	
Category8	
Category9	
Category10	

Variable 6	**Mobile phone number**
Name (GUI ENG)	Mobile phone number
Name (database)	MobilePhoneNumber
UMLS ID	C1549617
Type	Numeric
Length max char	50
Unit	
Group	User_db
Data entry	Textbox
Comments	
Terminology	
Index	NA
Category1	
Category2	
Category3	
Category4	
Category5	
Category6	
Category7	
Category8	
Category9	
Category10	

Variable 7	**Address**
Name (GUI ENG)	Address
Name (database)	Address
UMLS ID	C1442065
Type	String
Length max char	100
Unit	
Group	User_db
Data entry	Textbox
Comments	
Terminology	
Index	NA
Category1	
Category2	
Category3	
Category4	
Category5	
Category6	
Category7	
Category8	
Category9	
Category10	

Variable 8	**Patient ID**
Name (GUI ENG)	Patient ID
Name (database)	PatientID
UMLS ID	C2706990
Type	String
Length max char	
Unit	
Group	Patient_db
Data entry	Autogenerate
Comments	
Terminology	
Index	NA
Category1	
Category2	
Category3	
Category4	
Category5	
Category6	
Category7	
Category8	
Category9	
Category10	

Variable 9	**Patient age**
Name (GUI ENG)	Patient age
Name (database)	PatientAge
UMLS ID	C3166952
Type	Numeric
Length max char	25
Unit	In months if age < 1 year and in years if age >= 1 year
Group	Patient_db
Data entry	Textbox
Comments	
Terminology	
Index	Patient_age
Category1	Integer
Category2	Unknown
Category3	
Category4	
Category5	
Category6	
Category7	
Category8	
Category9	
Category10	

Variable 10	**Patient gender**
Name (GUI ENG)	Patient gender
Name (database)	PatientGender
UMLS ID	C0079399
Type	String
Length max char	25
Unit	
Group	Patient_db
Data entry	Dropdown
Comments	
Terminology	
Index	PatientGender
Category1	Female
Category2	Male
Category3	Unknown
Category4	
Category5	
Category6	
Category7	
Category8	
Category9	
Category10	

Variable 11	**Patient weight**
Name (GUI ENG)	Patient weight
Name (database)	PatientWeight
UMLS ID	C0005910
Type	Numeric
Length max char	25
Unit	In kilograms
Group	Patient_db
Data entry	Dropdown
Comments	
Terminology	
Index	Patient_weight
Category1	Integer
Category2	Unknown
Category3	
Category4	
Category5	
Category6	
Category7	
Category8	
Category9	
Category10	

Variable 12	**Time since injury**
Name (GUI ENG)	Time since injury
Name (database)	TimeSinceInjury
UMLS ID	C0449246
Type	String
Length max char	
Unit	
Group	Patient_db
Data entry	Autocalculated
Comments	
Terminology	
Index	TimeSinceInjury
Category1	1-24 hours ago
Category2	24-72 hours ago
Category3	72 hrs-1 week ago
Category4	>than 1 week ago
Category5	Unknown
Category6	
Category7	
Category8	
Category9	
Category10	

Variable 13	**Cause of injury**
Name (GUI ENG)	Cause of injury
Name (database)	CauseOfInjury
UMLS ID	C2025548
Type	String
Length max char	
Unit	
Group	Patient_db
Data entry	Dropdown
Comments	
Terminology	ICD10
Index	Cause_of_injury
Category1	Fire/flame
Category2	Hot object
Category3	Hot water
Category4	Chemical
Category5	Frozen object
Category6	Electricity
Category7	Other
Category8	Unknown
Category9	
Category10	

Variable 14	**Total burn surface area**
Name (GUI ENG)	Total burn surface area
Name (database)	TBSA
UMLS ID	C0433297
Type	String
Length max char	
Unit	
Group	Patient_db
Data entry	Dropdown
Comments	
Terminology	
Index	Total_burn_surface_area
Category1	
Category2	
Category3	
Category4	
Category5	
Category6	
Category7	
Category8	
Category9	
Category10	

Variable 15	**Body part**
Name (GUI ENG)	Body part
Name (database)	BodyPart
UMLS ID	C0229962
Type	String
Length max char	
Unit	
Group	Patient_db
Data entry	Checkbox
Comments	
Terminology	
Index	Body_parts
Category1	Head
Category2	Chest
Category3	Upper arm Across joint
Category4	Upper arm No across joint
Category5	Lower arm Across joint
Category6	Lower arm Not across joint
Category7	Hand Across joint
Category8	Hand Not across joint
Category9	Abdomen
Category10	Genitals
Category11	Upper leg Across joint
Category12	Upper leg Not across joint
Category13	Lower leg Across joint
Category14	Lower leg Not across joint
Category15	Foot

Variable 16	**Side**
Name (GUI ENG)	Side
Name (database)	Side
UMLS ID	C0441987
Type	String
Length max char	
Unit	
Group	Patient_db
Data entry	Dropdown
Comments	
Terminology	
Index	Side
Category1	Right
Category2	Left
Category3	Anterior (front)
Category4	Posterior (back)
Category5	
Category6	
Category7	
Category8	
Category9	
Category10	

Variable 17	**Pre-Existing Condition**
Name (GUI ENG)	Pre-Existing Condition
Name (database)	PreExistingCondition
UMLS ID	C0521987
Type	String
Length max char	
Unit	
Group	Patient_db
Data entry	Checkbox
Comments	
Terminology	
Index	Pre-Existing Condition
Category1	Diabetes
Category2	HIV
Category3	Tuberculosis
Category4	Ischaemic heart disease
Category5	Cancer
Category6	Nuntrintional deficiency
Category7	No/poor immunization history
Category8	Other
Category9	
Category10	

Variable 18	**Additional comments**
Name (GUI ENG)	Additional comments
Name (database)	AdditionalComments
UMLS ID	C1830770
Type	String
Length max char	
Unit	
Group	Patient_db
Data entry	Textbox
Comments	
Terminology	
Index	
Category1	
Category2	
Category3	
Category4	
Category5	
Category6	
Category7	
Category8	
Category9	
Category10	

Variable 19	**Where to treat**
Name (GUI ENG)	Where to treat
Name (database)	WhereToTreat
UMLS ID	C2585021
Type	String
Length max char	
Unit	
Group	Management_db
Data entry	Options
Comments	
Terminology	
Index	Where_to_treat
Category1	Discharge
Category2	Review in own facility
Category3	Admit own facility
Category4	Refer local referral hospital
Category5	Refer burn unit
Category6	
Category7	
Category8	
Category9	
Category10	

Variable 20	**Fluid Therapy**
Name (GUI ENG)	Fluid Therapy
Name (database)	FluidTherapy
UMLS ID	C0016286
Type	String
Length max char	
Unit	
Group	Management_db
Data entry	Checklist
Comments	
Terminology	
Index	Fluids
Category1	Glucose (basic needs)
Category2	Colloids
Category3	Ringerlactate
Category4	Blood
Category5	
Category6	
Category7	
Category8	
Category9	
Category10	

Variable 21	**Volume of fluid**
Name (GUI ENG)	Volume of fluid
Name (database)	VolumeOfFluid
UMLS ID	C0449971
Type	Numeric
Length max char	
Unit	ml
Group	Management_db
Data entry	Textbox
Comments	
Terminology	
Index	
Category1	
Category2	
Category3	
Category4	
Category5	
Category6	
Category7	
Category8	
Category9	
Category10	

Variable 22	**Drugs**
Name (GUI ENG)	Drugs
Name (database)	Drugs
UMLS ID	C0013227
Type	String
Length max char	
Unit	
Group	Management_db
Data entry	Textbox
Comments	
Terminology	
Index	
Category1	
Category2	
Category3	
Category4	
Category5	
Category6	
Category7	
Category8	
Category9	
Category10	

Variable 23	**Drug dose**
Name (GUI ENG)	Drug dose
Name (database)	DrugDose
UMLS ID	C0678766
Type	String
Length max char	
Unit	
Group	Management_db
Data entry	Textbox
Comments	
Terminology	
Index	
Category1	
Category2	
Category3	
Category4	
Category5	
Category6	
Category7	
Category8	
Category9	
Category10	

Variable 24	**Burn dressings**
Name (GUI ENG)	Burn dressings
Name (database)	BurnDressings
UMLS ID	C0180518
Type	String
Length max char	
Unit	
Group	Management_db
Data entry	Textbox
Comments	
Terminology	
Index	
Category1	
Category2	
Category3	
Category4	
Category5	
Category6	
Category7	
Category8	
Category9	
Category10	

Variable 25	**Specific instructions**
Name (GUI ENG)	Specific instructions
Name (database)	SpecificInstructions
UMLS ID	C1442085
Type	String
Length max char	
Unit	
Group	Management_db
Data entry	Textbox
Comments	
Terminology	
Index	
Category1	
Category2	
Category3	
Category4	
Category5	
Category6	
Category7	
Category8	
Category9	
Category10	
